# Emerging therapies in primary sclerosing cholangitis: pathophysiological basis and clinical opportunities

**DOI:** 10.1007/s00535-020-01681-z

**Published:** 2020-03-28

**Authors:** Mette Vesterhus, Tom Hemming Karlsen

**Affiliations:** 1 Norwegian PSC Research Center, Department of Transplantation Medicine, Division of Surgery, Inflammatory Medicine and Transplantation, Oslo University Hospital Rikshospitalet, Nydalen, Postboks 4950, 0424 Oslo, Norway; 2 Department of Medicine, Haraldsplass Deaconess Hospital, Bergen, Norway; 3 Institute of Clinical Medicine, University of Oslo, Oslo, Norway; 4 Section of Gastroenterology, Department of Transplantation Medicine, Oslo University Hospital, Oslo, Norway

**Keywords:** Primary sclerosing cholangitis, Therapy, Study design, Alkaline phosphatase

## Abstract

Primary sclerosing cholangitis (PSC) is a progressive liver disease, histologically characterized by inflammation and fibrosis of the bile ducts, and clinically leading to multi-focal biliary strictures and with time cirrhosis and liver failure. Patients bear a significant risk of cholangiocarcinoma and colorectal cancer, and frequently have concomitant inflammatory bowel disease and autoimmune disease manifestations. To date, no medical therapy has proven significant impact on clinical outcomes and most patients ultimately need liver transplantation. Several treatment strategies have failed in the past and whilst prescription of ursodeoxycholic acid (UDCA) prevails, controversy regarding benefits remains. Lack of statistical power, slow and variable disease progression, lack of surrogate biomarkers for disease severity and other challenges in trial design serve as critical obstacles in the development of effective therapy. Advances in our understanding of PSC pathogenesis and biliary physiology over recent years has however led to a surge of clinical trials targeting various mechanistic compartments and currently raising hopes for imminent changes in patient management. Here, in light of pathophysiology, we outline and critically evaluate emerging treatment strategies in PSC, as tested in recent or ongoing phase II and III trials, stratified per a triad of targets of nuclear and membrane receptors regulating bile acid metabolism, immune modulators, and effects on the gut microbiome. Furthermore, we revisit the UDCA trials of the past and critically discuss relevant aspects of clinical trial design, including how the choice of endpoints, alkaline phosphatase in particular, may affect the future path to novel, effective PSC therapeutics.

## Introduction

Primary sclerosing cholangitis (PSC) is a rare and slowly progressive liver disease with strong genetic and clinical associations with autoimmunity and characterized by multi-focal inflammatory and fibrotic bile duct strictures leading to fluctuating cholestasis, cirrhosis, and ultimately end-stage liver disease [1]. Diagnosis is based on the demonstration of characteristic cholangiographic bile duct findings in the setting of elevated alkaline phosphatase (ALP) and following exclusion of differential diagnoses [e.g. immunoglobulin G4 (IgG4) associated sclerosing cholangitis]; magnetic resonance cholangiography (MRC) is the method of choice. In many patients, ALP fluctuates, and may be normal. Subgroups of PSC patients exist and must be accounted for in clinical practice and outcome assessments (e.g. small-duct PSC, PSC with ulcerative colitis or Crohn’s colitis, PSC with elevated IgG4 [2–4]), likely to represent, however, variant forms of the same pathophysiological entity.

Considered a rare disease affecting around 1/10,000 in the most prevalent areas of Northern Europe and the US, PSC occurs at all ages although typically diagnosed in younger adults 30–40 years of age with inflammatory bowel disease (IBD; in 70–80%, 50–60% or 20–30% in Northern Europe and USA, Southern Europe, and Asia, respectively). Patients with PSC carry an exceptionally high risk of hepatobiliary and colorectal malignancy with a cumulated risk of cholangiocarcinoma (CCA) approaching 20% at 30 years in some patient series [5]. Many patients develop recurrent bacterial cholangitis, biliary sludge or gallstones, or symptomatic biliary strictures even without such complications (often coined “dominant strictures”) that may profit from endoscopic treatment with balloon dilatation [6, 7]. Because of the complications and co-morbidities, although rare, PSC represents a significant burden for patients as well as for specialized health services. Critical unmet needs include lack of effective medical therapy, lack of tools for early detection of CCA, and reliable biomarkers for prognostication in the setting of a highly variable disease.

There is currently no effective medical therapy with benefit for clinical outcomes in patients with PSC. Ursodeoxycholic acid (UDCA), whilst considered standard-of-care in primary biliary cholangitis (PBC), has failed to show significant and consistent effects on transplant-free survival in PSC [8]. Prescription is still widespread, and often a trial period of 3–6 months is employed, after which the decision for continued UDCA treatment is done based on biochemical response and potential symptomatic benefits (reduced pruritus) [9]. High doses of UDCA (> 20 mg/kg/day) should be avoided [10]. In regions where UDCA prescription is less prevalent (e.g. Northern Europe and the US), patients are currently left with symptomatic measures (e.g. to control pruritus and osteopenia) and clinical surveillance only, with endoscopic therapy and ultimately liver transplantation as invasive treatment options for clinically significant biliary strictures and end-stage liver disease, respectively.

In high-prevalence areas like Scandinavia, PSC is a major indication for liver transplantation [11]. Mortality is increased fourfold compared to the general population, partly due to end-stage liver disease; however, more than 40% of PSC deaths have been attributed to cancer development [5]. Current tools for early detection of CCA perform poorly; however, annual screening by hepatobiliary imaging and full ileocolonoscopy is recommended by international guidelines [12]. There is currently a trend away from annual ultrasound-based screening for gallbladder polyps (and hepatocellular cancer in cirrhotic patients) towards the use of magnetic resonance imaging (MRI) and MRC as the annual screening imaging modality of choice [13, 14], but the full utility of various screening modalities for early cancer detection in PSC awaits prospective validation [15].

## Pathophysiological basis of therapy

A major challenge in identifying effective therapeutic approaches is that a proven conceptual framework is still lacking for PSC pathogenesis. The pathogenesis of PSC currently appears complex, many-facetted and with an incomplete understanding of primary versus secondary processes, leaving critical knowledge gaps in the selection of potential therapeutic targets [1]. Both environmental and genetic causes are believed to play a part in establishing pathways currently thought to drive pathogenesis, through avenues involving toxic effects of bile due to altered bile acid composition and cholestasis [16], factors related to the gut microbiota [17], as well as autoimmunity [18], all contributing to inflammation, fibrosis, and carcinogenesis in PSC.

There is a strong genetic evidence for autoimmune susceptibility as a basis for interest in immune-modulating therapy. Genome-wide association studies (GWAS) have identified more than 20 genetic risk loci [19–25], clearly establishing PSC as an autoimmune disease as seen from the genetic perspective [18]. Furthermore, data have proposed that pathogenic T-cells originating from colonic and small bowel activation, may subsequently migrate to the liver driven by an overlapping expression in the gut and the liver of relevant lymphocyte homing components including the α4β7 integrin and mucosal vascular addressin cell adhesion molecule 1 (MAdCAM-1) [26]. In the liver, these recruited lymphocytes have been suggested to involve in the biliary inflammation leading to apoptosis and necrosis of cholangiocytes, and with time tissue fibrosis [27, 28].

Gut-derived antigens presented by PSC-associated human leukocyte antigen (HLA) variants to the T cell receptor (TCR) may also contribute to adaptive immune responses in the portal areas by means of molecular mimicry [29–31]. Possibly, gut leakage of pro-inflammatory bacterial products (e.g. lipopolysaccharides, LPS) also contributes by involving innate immune responses [32–35]. Furthermore, a series of studies now strongly indicate that the gut microbiota may be involved in PSC pathogenesis [17, 36–40] giving rise to clinical trials involving fecal transplantation, non-absorbable antibiotics, and other means of manipulating the gut microbiome in patients [17, 41–43]. In the bile ducts, bacterial, and fungal colonization may follow cholestasis and endothelial damage, through the establishing of a pathogenic biliary microbiota further propagating inflammation and intercurrent infections [44].

Toxic effects of bile upon cholangiocytes [32, 45], due to cholestasis, or primary or secondary changes in bile composition as part of disease processes in the bile ducts or colon [46–50], or impairment of protective means (e.g. the so-called “bicarbonate umbrella”) [51], may contribute to biliary inflammatory and fibrotic processes. This “toxic bile hypothesis” has inspired a broad portfolio of compounds aimed at manipulation of bile acids, cholestasis and regulators of bile acid metabolism, nuclear receptors included [52, 53].

Regardless of the order of mentioned pathophysiological events or the initiating factors, a final common pathway of cellular crosstalk leads to activation of stellate cells (and possibly portal myofibroblasts) with fibrosis, collagen deposition and generation of the scar tissue causing the bile duct strictures [54, 55], processes revealing targets for antifibrotic therapy [56–58]. The relative importance of the many elements believed to contribute to PSC development and progression is unknown and may vary between subgroups of patients and depending on disease stage (early disease likely to yield other opportunities for therapy than late stage disease).

**Fig. 1:**
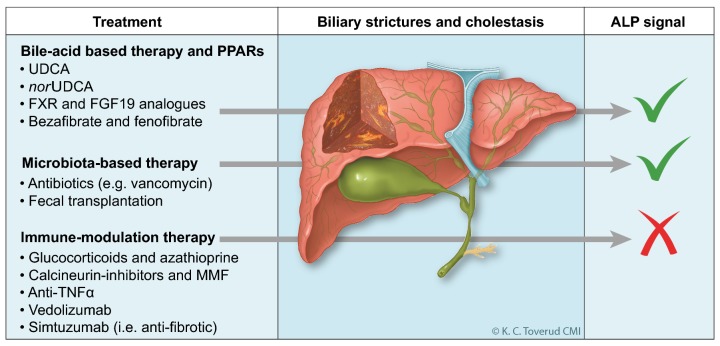
Summary of therapeutic approaches in primary sclerosing cholangitis (PSC). Numerous smaller trials have been performed to assess the clinical efficacy of wide spectrum of drugs in PSC (left panel). None of the categories of compounds tested has shown significant impact on the progression of biliary strictures (center panel) and the development of end-stage liver disease. Several therapeutics affecting bile acid physiology and the gut microbiota influence alkaline phosphatase (ALP) and other potential markers of disease severity (right panel), but the clinical importance of this phenomenon remains to be firmly established in prospective patient assessments. Paradoxically, despite strong genetic and clinical associations with autoimmune co-morbidities and a strong mechanistic rationale, no convincing clinical efficacy has been observed for immunosuppressive or anti-fibrotic drugs to date. *PPAR* peroxisome  proliferator-activated receptor, *UDCA* ursodeoxycholic acid, *FXR* farnesoid X receptor, *FGF19* fibroblast growth factor 19, *MMF* mycophenolate mofetil, *TNFα* tumor necrosis factor alpha. Printed with permission from Kari C. Toverud

Following the many developments in our understanding of PSC pathogenesis, the most prominent feature of the PSC research field these days is the emergence of a variety of clinical trials (Fig. 1). This new situation has raised hopes for the emergence of effective therapeutics in PSC, showing little reminiscence of the scenario 5–10 years ago when there was little or no clinical trial activity. However, past experiences and the reasons for the failures of UDCA to show effects on clinical outcomes (and even increased mortality in the high-dose [28–30 mg/kg/day] UDCA trial), despite promising effects on hepatic biochemistries and prognostic scores, warrants consideration and caution in our evaluation of results from the ongoing trials [59]. The highly variable natural course of the disease with fluctuating symptoms and laboratory tests as well as its rarity and relatively slow progression causing low event-rates in clinical trials altogether complicate study design and the evaluation of results, and improved surrogate endpoints to tackle this situation are highly warranted [60, 61].

On the basis of these reflections, in the following we will outline the spectrum of emerging therapies, categorized by their plausible pathophysiological foundation. We will critically evaluate the current evidence base for these new therapeutic options and discuss how trial design may be optimized and improved to better allow us to achieve reliable results on which regulatory and clinical decision-making can be based.

## Therapeutic approaches

### Bile acid therapeutics

The term “cholestatic liver disease” is ambiguous, and may imply cholestasis both as cause and effect for hepatocellular and biliary changes observed in a variety of liver diseases. Obstructive cholestasis occurs in PSC as a consequence of biliary strictures, and bile acid toxicity has been argued to be a critical component in the development of progressive liver disease. Under the “toxic bile hypothesis”-model for PSC, it may also be argued that bile acids serve as initiating factors for the inflammation and fibro-obliterative changes to the bile ducts, either because of changes to bile composition, or to deficiencies in protective or compensatory mechanisms, the so-called “biliary bicarbonate umbrella” included. Bile formation is a complex physiological process, also involving protective mechanisms throughout the exposed surfaces of the biliary tract. Driven by the cystic fibrosis transmembrane conductance regulator (CFTR) mediated chloride secretion and chloride/bicarbonate anion exchanger type 2 (AE2) [51], cholangiocytes secrete a bicarbonate rich fluid contributing about 25% of the daily bile production. The bicarbonate is concentrated at the apical surface of the biliary epithelium, presumed to form a protective layer above the cholangiocytes, whereby deficient protection might lead to or aggravate biliary disease.

During cholestasis, compensatory mechanisms aim to alleviate the potential toxic side effects of bile components, bile acids in particular [62, 63]. The process is orchestrated by a family of quite promiscuous (i.e. having relatively broad ligand specificities) nuclear receptors for which bile acids also can serve as activating ligands, most notably the farnesoid X receptor (FXR) [64], the pregnane X receptor (PXR) [65], and the vitamin D receptor [66]. Contributions to the orchestrating efforts comes from related nuclear receptors with differing specificities, e.g. small heterodimer partner (SHP), the constitutive androstane receptor (CAR), peroxisome proliferator-activated receptor alpha (PPARα) and the glucocorticoid receptor, as reviewed elsewhere [62, 67]. Principle effects cover five main areas: (a) inhibition of bile acid synthesis [through cytochrome P 7A1 (CYP7A1)], (b) enhancement of detoxification (through CYPs, sulfotransferases and glucuronosyltransferases), (c) reduced basolateral bile acid uptake (mainly through downregulation of Na^+^-taurocholate cotransporting polypeptide [NTCP]), (d) increased basolateral (mainly through upregulation of solute carrier organic anion transporter family member 3A1 [SLCO3A1], organic solute transporter alpha and beta [OSTα/β] and multidrug resistance-associated protein 4 [MRP4]) and apical (through bile salt export pump [BSEP]) bile acid efflux, (d) pleiotropic effects of involved nuclear receptors on various inflammatory, apoptotic and fibrotic pathways.

The logic behind bile acid based therapies in PSC is thus to target unspecific (e.g. choleresis and bicarbonate secretion), specific (e.g. FXR activation) or pleiotropic (e.g. inflammation, apoptosis or fibrosis) aspects relating to bile acid physiology, resulting in enhanced protection and reduced harm from the intrinsic toxicity of bile acids, bile acid metabolites, and other bile constituencies. Interest in bile acid based therapy in PSC was however sparked long before many of these biological insights, by the success of UDCA in the treatment of PBC. Arguing against primary roles of bile acid toxicity in PSC initiation, GWAS revealed no susceptibility loci that clearly harbor genes potentially involved in bile acid homeostasis (with *TGR5* on chromosome 2 and *HDAC7* on chromosome 12 possible exceptions), and there is no data to support involvement of genes causing Mendelian cholestasis syndromes (e.g. multidrug resistance protein 3 [*MDR3*] and *BSEP*) in PSC. This probably indicates that bile acid toxicity and reduced cholangiocyte protection during cholestasis are rather than  for initiation involved in downstream, common pathways and disease progression, still rendering the processes as attractive therapeutic targets.

#### Ursodeoxycholic acid

UDCA is the most extensively studied and most frequently prescribed pharmaceutical agent in the treatment for PSC. UDCA is a hydrophilic bile acid which, in moderate doses, is thought to exert its mechanisms of action mainly through protective effects on cholangiocytes by reducing hydrophobicity and toxicity of bile via the stimulation of hepatobiliary secretion, and a direct effect on adaptive immunity e.g. by inhibiting dendritic cells, but the exact mechanisms by which UDCA exerts its effects have not been finally defined [68]. It is not, however, an FXR agonist.

Enthusiasm was high for UDCA as initial research indicated positive effects in PSC (Table 1). Five early placebo-controlled trials of UDCA at doses between 13 and 15 mg/kg/day showed effect on primary endpoints defined as reduction in ALP and other liver enzymes or reduction in the PSC-specific Mayo risk score [69–74]. However, sample sizes were small (*n* = 6, 10, 13, 20 and 51 patients in treatment groups, respectively) and statistical power insufficient, limiting the validity of the results. Furthermore, these studies were flawed by short duration (3, 12, 24 and 24 months and mean 2.2 years, respectively) compared to the natural history of PSC, evolving over decades.

**Table 1:** Results and effects on ALP of UDCA in PSC in therapeutic trials

References	Year	*N*	Design	Lab inclusion criteria	Results—ALP	Other results
Beuers et al. [70]	1992	14	12 months Treatment group: *N* = 6 (one of these was excluded at 6 months—> *n* = 5 in analyses)	ALP > 1.5 × ULN Bilirubin > 15 mg/dL	Reduction in ALP (− 67%) in UDCA group compared with placebo group; ΔALP − 72.6% in UDCA group compared to baseline; all 5 patients in UDCA group compared to 3/7 patients in the placebo group achieved ALP reduction	Reduced GGT (− 53%), bilirubin (− 50%) and ALT (− 36%) compared with placebo group
Lindor et al. [71]	1997	102	2 years (at least 3 months; median follow-up 2.2 years) Double-blind RCT Multicenter Treatment group: *N* = 51	ALP > 1.5 × ULN	ΔALP − 40.6% (UDCA group) compared to baseline, compared with ΔALP − 6.1% (placebo group)	ΔAST − 43.3% compared to baseline No significant effect on primary end-points (death, OLT, histology)
Mitchell et al. [72]	2001	26	2-years Double-blind RCT	Stable liver biochemistry for 3 months prior to entry; cholestatic liver enzyme pattern	Reduction in ALP (− 45.4%) compared with baseline	ΔGGT − 62.6% compared with baseline Reduced cholangiographic findings Reduction in histological stage using Modified Histological Activity Index after Ishak in the UDCA group: inflammation ↓ *n* = 5, unchanged *n* = 4, ↑ *n* = 2; stage ↓ one stage *n* = 3, unchanged *n* = 6, ↑ (progression) *n* = 2
Harnois et al. [196]	2001	30	12 months Pilot open-label study, compared to UDCA low-dose and placebo groups of a previous RCT Treatment group: *N* = 30	ALP > 1.5 × ULN	ALP reduction of > − 50% compared to baseline was achieved by 38% at 12 months Reduction in ALP (− 45.2%)	Reduction in AST (− 52.2%) Reduction in bilirubin (− 44.4%) in *n* = 11 with abnormal bilirubin at baseline Reduction in Mayo risk score was greater in UDCA high-dose group compared with reduction in the placebo and UDCA low-dose groups in a previous study (− 0.542 ± 0.15 vs 0.167 ± 0.09 and − 0.303 ± 0.12, respectively
Olsson et al. [8]	2005	219	5 years RCT Multicenter	No	A non-significant trend towards ALP reduction with ΔALP ca 0.3 µkat at 6 months and stable thereafter in UDCA group compared to no change in placebo group	No effect on death, OLT or CCA
Lindor et al. [10]	2009	149	6 years Treatment group: *N* = 76	ALP > 1.5 × ULN	Reduction in median ALP (− 48.5%) compared with baseline in UDCA group at 36 months (however *n* = 73 at baseline *n* = 53 at 36 months), − 25% in placebo group	Terminated at 6 years as worse outcome in treatment group for death or OLT

*ALP* alkaline phosphatase, *ALT* alanine transferase, *CCA* cholangiocarcinoma, *GGT* gamma-glutamyltransferase, *MELD* model for end-stage liver disease, *OLT* orthoptic liver transplantation, *RCT* randomized controlled trial, *UDCA* ursodeoxycholic acid, *ULN* upper limit of normal

Three randomized placebo-controlled trials (RCT) of UDCA in PSC have investigated clinical outcome parameters as the primary outcome, all failing to prove statistically significant beneficial effects on liver transplantation-free survival or cholangiocarcinoma [8, 10, 71]. Follow-up ranged from 2 to 5 years. The earlier of these trials succeeded in enrolling the predefined number of patients (*n* = 51 for UDCA and placebo groups, respectively), estimated to yield 70% power to detect a hazard ratio of 2.0 (placebo:UDCA) given an estimated survival free of  treatment failure of 3.3 years in this panel of patients with advanced liver disease. However, at 2 years, treatment failure had occurred in 16/31 versus17/32 in the UDCA and placebo groups, respectively, with 9 vs 8 liver transplantations and 4 vs 3 deaths (deaths not responsible for failure), leading to the conclusion of no treatment effect and speculations that this was due to advanced disease stage [71].

A major problem for the other two UDCA trials was their failure to include a sufficient number of patients to reach the pre-defined power thresholds. Out of 455 PSC patients who were screened for a high-dose UDCA trial, only 149 entered the study (*n* = 76 vs 74 in UDCA vs placebo groups), with 6 later withdrawals in the treatment group, with importantly the main indication for exclusion/non-eligibility being inadequate ALP elevation [10]. Sample size calculations based on an expectation that 35% of patients would reach the primary endpoint within 5 years estimated that *n* = 149 patients would yield 80% power to detect a 50% reduction in event-rate in UDCA compared to placebo with 5% level of significance, with a co-primary endpoint including death, liver transplantation, meeting minimal listing criteria, development of varices, cholangiocarcinoma or progressing to cirrhosis. However, event-rates for death and liver transplantation were low [5 vs 3 deaths and 11 vs 5 transplantations in the UDCA (*n* = 76) and placebo (*n* = 74) groups, respectively] [10].

Even the largest multicenter RCT to date, including *n* = 219 patients, of which 97 patients treated with UDCA (17–23 mg/kg/day) with 5 year follow-up, failed to recruit the predefined required number of patients (*n* = 346) to detect a statistically significant difference (80% power to detect a 50% reduction in event-rate in UDCA compared to placebo with 5% level of significance) in the primary endpoint [8]. Only 11/101 vs 7/97 patients in the placebo and UDCA groups, respectively, reached the co-primary endpoint of liver transplantation or death (*P* = 0.37). No significant effect of UDCA was demonstrated in this study on either liver enzymes, cholangiocarcinoma or transplantation-free survival. Although the likelihood of a type II error is high, the continued failure to demonstrate any effect in the 15 year follow-up study (no difference between UDCA and placebo group regarding the primary endpoint of cancer-free survival, *P* = 0.73; a total of 8 and 6 patients died in the UDCA and placebo groups, respectively) supports the conclusion of the original paper [75]. Four meta-analyses have failed to demonstrate benefit on survival of UDCA in PSC [76–79].

Based on physiological data on a dose-dependent increase in UDCA enrichment of the bile of patients with PSC [43–47% at normal doses (10–17 mg/kg/day); 56–59% at high-dose (18–32 mg/kg/day)] [80], pilot assessments were performed that indicated enhanced efficacy of high-dose UDCA prescription in PSC [72, 81]. As a follow-up to this, a multicenter high-dose (28–30 mg/kg/day) UDCA clinical trial was carried out, surprisingly observing an increase in clinical endpoints including liver transplantation and death, colorectal dysplasia, and development of esophageal varices [10, 59, 82]. In consequence, the subsequent American Association for the Study of Liver Diseases (AASLD) clinical practice guidelines advised against the use of UDCA as medical therapy in PSC [83], whereas the European Association for the Study of the Liver (EASL) guidelines conclude that no specific recommendation for the general use of UDCA in PSC may be made based on the inadequate present data [12]. The mechanisms explaining the detrimental effects of high-dose UDCA have not been fully elucidated, but may be partly linked to the increased amount of unabsorbed UDCA delivered to the colon and changes in microbial bile acid metabolism [47].

The debate surrounding the high-dose trial led to a dramatic drop in UDCA prescription for PSC, in the US particularly, and even loss of on-label prescription in some countries. As elaborated below, the UDCA trials should thus be scrutinized for learning experiences of relevance to ongoing and future trials, given the persisting emphasis on ALP as a surrogate biomarker for treatment effect. Critical questions must be asked: is reduction in ALP a valid endpoint in trials for PSC and what is a clinically significant reduction in ALP in PSC, over and above the natural ALP fluctuations known to occur? In scientific terms, the question for UDCA in PSC is still open; a well-powered, adequately designed clinical trial has not yet been performed—and likely never will be. What seems certain is that high-dose UDCA should be avoided, but for lower doses (10–15 mg/kg/day) current prescription is left at the discretion of the individual physician—a situation clearly not acceptable for a proposed first-in-line therapeutic in a devastating disease.

#### *nor*Ursodeoxycholic acid

24-Norursodeoxycholic acid (*nor*UDCA) is a synthetic side chain-shortened UDCA derivative, which is resistant to amidation and undergoes cholehepatic shunting [84]. *Nor*UDCA appears to enhance general resistance to bile acid induced biliary injury, partially via a bile-acid dependent bicarbonate-rich choleresis created through stimulation of canalicular flow, proposedly reinforcing the “bicarbonate umbrella” [85, 86]. Furthermore, pleiotropic effects on inflammatory, apoptotic and fibrotic pathways over and above the choleresis likely contribute to the pre-clinical effects [87, 88]. In contrast to UDCA, *nor*UDCA is secreted into bile in an unconjugated, glucuronidated form and its metabolite, nor-lithocholate, does not accumulate in hepatocytes or cause hepatotoxicity in animal models. This may be an important point, as markedly elevated levels of hepatotoxic lithocholic acid (LCA) was observed in the treatment arm in post-hoc analyses of serum bile acid composition from PSC patients in the high-dose UDCA trial, possibly contributing to the increased rates of adverse outcomes.

A multicenter phase II clinical trial including 161 PSC patients without concomitant UDCA therapy demonstrated dose-dependent serum ALP reduction (12.3%, 17.3% or 26.0% vs placebo, respectively) during a 12-week treatment with *nor*UDCA 500 mg/day, 1000 mg/day, 1500 mg/day or placebo (Table 2). Moreover, *nor*UDCA induced a similar dose-dependent reduction in serum transaminases. Importantly, *nor*UDCA showed an excellent safety profile similar to placebo and pruritus-rates were not different from placebo. Based on these promising findings, a Phase III study (NUC5/PSC) is ongoing (Table 3).

**Table 2:** Results of non-UDCA therapeutic trials in PSC

Therapy	References	*N*	Design	Lab inclusion criteria	Primary endpoint	Result ALP	Other results
*Therapy targeting bile acids*							
norUDCA UDCA derivative	Fickert et al. [197]	161	RCT Multicenter Phase II 12 weeks	Bilirubin < 3.0 mg/dL	ΔALP at 12 weeks	Significant dose-dependent reductions in ALP; ΔALP (compared to placebo) − 12.3%, − 17.3% and − 26.0% in the 500, 1000 and 1500 mg treatment groups	Favorable safety profile (no increase in pruritus)
NGM282 FGF-19 analogue	Hirschfield et al. [107]	62	RCT Phase II 12 weeks	No	ΔALP at 12 weeks	No significant change in ALP	Reduced BA Improved (reduced) fibrosis markers ELF test and PRO-C3
Obeticholic acid (OCA) FXR agonist AESOP trial	Kowdley et al. [100]	76	RCT Phase II 24 weeks	ALP ≥ 2.0*ULN Bilirubin < 2.5*ULN	ΔALP at 24 weeks	Significant reduction in ALP in the 5–10 mg treatment arm compared to placebo; ΔALP − 25% from baseline in the 5–10 mg treatment arm compared to ΔALP − 4.8% in placebo group; ΔALP − 14% vs − 25% in patients with and without UDCA at baseline in the 5–10 mg OCA arm	Increased pruritus; pruritus (severe pruritus) reported in 46% (8%), 60% (16%) and 67% (41%) in placebo, 1.5–3 mg and 5–10 mg groups; *n* = 15 drop-outs prior to 24 week assessment
LUM001/maralixibat ASBT inhibitor CAMEO trial	Completed; Results at clinicaltrials.gov	27	Open label pilot 14 weeks	ALT and AST ≤ 5*ULN	Δbile acid levels at 14 weeks	No reduction in ALP	ΔBA − 14.8 (− 38%)
*Therapy targeting PPAR*							
Bezafibrate 400 mg/day	Mizuno et al. [121]	7	Open-label pilot 6 months	ALP > 1.5 × ULN	ΔALP at 6 months	ALP reduction with about 40% in 3/7 patients at 6 months	
Bezafibrate 400 mg/day	Mizuno et al. [122]	11	Open-label pilot 12 weeks		ΔALP at 12 weeks	ALP reduction at 12 weeks, ALP increase subsequent to treatment cessation	
Bezafibrate 400 mg/day or fenofibrate 200 mg/day	Lemoinne et al. [123]	20	Retrospective study	ALP > 1.5 × ULN on UDCA	ΔALP	Reduced ALP after at least 6 months; 40% reached ALP < 1.5 × ULN	Reduced ALT and pruritus
Fenofibrate	Dejman et al. [124]	8	Open label pilot 6 months	ALP > 1.5 × ULN	ΔALP at 6 months	Significant ALP reduction: ΔALP − 43%	Reduced ALT No significant effect on Mayo risk score
*Therapy targeting gut microbiota*							
Vancomycin vs metronidazole	Tabibian et al. [137]	28	RCT Phase II-III Multicenter 12 weeks	ALP > 1.5*ULN	ΔALP at 12 weeks	Non-dose dependent ALP reduction in all 4 treatment arms (low vs high dose vancomycin or metronidazole)	
Vancomycin	Rahimpour et al. [138]	29	RCT 12 weeks	No	ΔMayo risk score	ALP reduction at 12 weeks; ΔALP − 18.2%	Reduced Mayo risk score
Metronidazole	Farkkila et al. 2004 [139]	80	RCT Phase III 36 months	No / not specified (possibly ALP or ALT > ULN)	ΔALP or other liver enzymes, Mayo risk score, symptoms or histology at 36 months	ALP reduction at 36 months; ΔALP − 52.4% vs − 37.7% in metronidazole + UDCA group vs UDCA + placebo group	Reduced Mayo risk score; higher proportion of patients showed histologic improvement of stage or grade
Minocycline	Silveira et al. [143]	16	Open-label pilot 12 months	ALP > 1.5*ULN	ΔALP at 12 months	ALP reduction at 12 months; ΔALP − 20%	Reduced Mayo risk score
Rifaximin	Tabibian et al. [140]	16	Open-label pilot 3 months	ALP > 1.5*ULN	50% ALP reduction at 3 months	No significant ALP reduction	No significant reduction in bilirubin, GGT, Mayo risk score
Fecal transplantation	Allegretti et al. [41]	10	Open-label pilot 24 weeks	ALP > 1.5*ULN	≥ 50% ALP reduction at week 24	30% (3/10) experienced a ≥ 50% decrease in ALP	
*Immune modulating therapy*							
All-trans retinoic acid	Assis et al. [115]	15	Open-label pilot 12 weeks	ALP > 1.5 × ULN on UDCA	ΔALP − 30% at 12 weeks	Non-significant ALP reduction; 3/15 achieved ≥ 30% ALP reduction	Reduced ALT and C4; ALT returned to pre-treatment values after washout period
Infliximab	Hommes et al. [167]	10	RCT 52 weeks	ALP > 2*ULN	≥ 50% ALP reduction at week 18	Failed to demonstrate effect in the *n* = 6 treatment group	No change in histologic stage or symptom scores
*Other or undefined targets*							
Cenicriviroc Anti-inflammatory effects (CCR2/CCR5 antagonist) PERSEUS trial	Completed, not published; results at clinicaltrials.gov	20	Open label Phase II 24 weeks	ALP > 1.5*ULN Bilirubin ≤ 2.0 mg/dL	ALP (%Δ)	50% (*n* = 10) of patients achieved ALP reduction to < 1.5*ULN at 24 weeks. Mean ALP reduction − 4.5% at 24 weeks. No patients achieved ALP normalization or 50% ALP reduction	
Curcumin Anti-inflammatory effects, upregulation of PPAR-γ?	Completed, not published	15	Phase I–II Open-label	ALP > 1.5*ULN	ALP 40% reduction or reduction to < 1.5*ULN	Results submitted to clinicaltrials.gov, but not posted	

*ALP* alkaline phosphatase, *ALT* alanine transferase, *ASBT* apical sodium-dependent bile acid transporter, *BA* bile acids, *C4* 7 Alpha-hydroxy-4-cholesten-3-one (marker of bile acid synthesis), *CCA* cholangiocarcinoma, *ELF* Enhanced Liver Fibrosis test, *FGF-19* fibroblast growth factor 19, *FXR* farnesoid X receptor, *GGT* gamma-glutamyltransferase, *PPAR* peroxisome proliferator-activated receptor, *PRO-C3* marker of type III collagen formation, *RCT* randomized controlled trial, *UDCA* ursodeoxycholic acid, *ULN* upper limit of normal

**Table 3:** Some currently registered and ongoing therapeutic trials in adult PSC

Therapy	Pathophysiologic target	Trial phase	Design	Lab inclusion criteria	Primary endpoint	Secondary endpoint
*Bile acid based therapy*						
*Nor*UDCA	UDCA derivative Unknown receptor	III	Double-blind RCT Multicenter	No (?)	ALP partial normalization	Histology
Cilofexor	FXR agonist	III	Double-blind RCT	ALT ≤ 8*ULN Bilirubin ≤ 2.0 mg/dL INR ≤ 1.4 Platelets ≥ 150,000	Histology	ΔALP Δother liver biochemistries ΔLSM (TE) + +
*Therapy targeting PPAR*						
Seladelpar	Selective PPAR-δ agonist	II	Double-blind RCT	ALP ≥ 1.5*ULN and < 8*ULN Bilirubin ≤ 2*ULN ALT and AST ≤ 5*ULN Platelets ≥ 140,000	ΔALP at 24 w	LTX ΔMELD Hepatic decompensating events HCC
Bezafibrate	PPAR-α agonist	III	Double-blind RCT	No	Proportion of patients reaching 50% reduction in itch intensity at 3 weeks	Δliver biochemistries Δautotaxin activity Δcholesterol, CK, creatinine
*Therapy targeting gut microbiota*						
Vancomycin	Antibiotic	III	Double-blind RCT Multicenter	ALP ≥ 1.5*ULN	ALP normalization at 6, 12, 18, 21, 24 months	ΔTE at 18 months
*Immune modulating therapy*						
Simvastatin	Immune modulating, receptor?	III	Double-blind RCT Multicenter	No	Overall survival; Listing for liver transplantation; Time to first varices bleeding or CCA, GBC, HCC	ΔALP Δbilirubin ΔMELD or ΔChild–Pugh MRCP progression ΔLSM (TE) or Δserum fibrosis markers Progression of symptoms, biliary dysplasia, colon cancer or dysplasia
Timolumab BTT1023 BUTEO trial	Anti-VAP-1 antibody	II	Open label	ALP > 1.5*ULN Stable ALP i.e. < 25% variation between screening visits 1 and 2	ALP 25% reduction by day 99	
All-trans retinoic acid	FXR/RXR complex activation	II	Open label	ALP ≥ 1.5*ULN	ΔALP at 24 w	ALP > 1.5*ULN ΔALT Δbile acids ΔELF test ΔLSM (TE)
Sulfasalazine	Immune modulating	II	Double-blind RCT Multicenter	ALP ≥ 1.67*ULN Bilirubin ≤ 3 mg/dL INR ≤ 1.4 Platelets ≥ 100,000 MELD ≤ 10	ALP ≥ 1.5 at 22 w	Δother liver biochemistries ΔMayo risk score Symptoms
Vidofludimus calcium	Blocks IL-17 production	II	Open label	ALP > 1.5*ULN Indirect bilirubin < 1.2 *ULN	ΔALP at 3 and 6 months	Δother liver biochemistries IL-17 and IFNγ levels at 3 and 6 months

*ALP* alkaline phosphatase, *ALT* alanine transferase, *CCA* cholangiocarcinoma, *ELF* Enhanced Liver Fibrosis test, *FXR* farnesoid X receptor, *GBC* gallbladder cancer, *HCC* hepatocellular cancer, *IFNγ* interferon gamma, *LSM* liver stiffness measurements, *MELD* model for end-stage liver disease, *PPAR* peroxisome proliferator-activated receptor, *RCT* randomized controlled trial, *RXR* retinoid X receptor, *TE* transient elastography, *ULN* upper limit of normal, *VAP-1* vascular adhesion protein-1

#### Steroidal FXR agonists

The nuclear bile acid receptor FXR has been implicated in human cholestatic conditions such as progressive familial intrahepatic cholestasis (PFIC) type 1 and intrahepatic cholestasis of pregnancy [89, 90]. Exemplified, two pairs of siblings with homozygous FXR truncation or deletion variants were reported, of which all four children suffered from severe, early-onset PFIC and liver failure before the age of two [91]. One of the key roles of FXR is downregulating CYP7A1, the rate-limiting enzyme in bile acid production. Negative feedback also affects bile acid turnover, directly through FXR activation in the liver and indirectly via FXR activation in the gut leading to downregulation of enterocyte apical sodium dependent bile acid transporter (ASBT) and increased fibroblast growth factor 19 (FGF19) secretion, which signals to hepatocytes via portal blood and hepatocyte fibroblast growth factor receptor 4 (FGFR4) activation [92]. Like several of the other bile acid receptors involved in the regulation of bile acid physiology, FXR directly or indirectly induce pleiotropic effects affecting inflammation and metabolism [93, 94]. Therapeutic activation of FXR and FGF19 is under heavy investigation in cholestatic and metabolic liver diseases, utilizing both bile-acid derivatives (e.g. obeticholic acid [OCA]) and small-molecular, synthetic FXR analogues (discussed below) (Tables 2, 3).

The selective FXR agonist OCA (6α-ethyl-chenodeoxycholic acid) was approved as second line treatment in PBC based on significant ALP reductions compared to placebo in one phase III trial (POISE; *n* = 217) and data from a 3-year open-label follow-up study were favorable [95–97]. Dose-dependent pruritus occurred in many patients, a particular problem for cholestatic patients in whom pruritus often occurs as a complication to disease. Also in non-alcohol related fatty liver disease (NAFLD), phase II and III efficacy signals have been reported [98, 99]. OCA is derived from the primary human bile acid chenodeoxycholic acid (an endogenous FXR ligand), and chemically modified to make it 100 times more potent.

The efficacy and safety of OCA in patients with PSC was evaluated in dose-finding RCT AESOP, using change in ALP from baseline as the primary outcome (Table 2) [100]. In this trial, treatment arms given OCA 1.5–3 mg or 5–10 mg in ALP-dependent titrating dose levels were compared to placebo. Results showed effect on the primary endpoint with a similar reduction in ALP of about 25% at 24 weeks for the two treatment arms; however, only OCA 5–10 mg achieved statistically significant increased ALP reduction compared to placebo (ΔALP  difference OCA-placebo − 83.4, *P* = 0.04; ca − 25% vs − 4.8%.

OCA was generally safe and well tolerated [100]. However, pruritus was reported as a side effect in both treatment arms but not in the placebo group in a dose-dependent pattern, despite exclusion of patients with severe pruritus at baseline and a higher proportion of patients in the placebo group compared to the 5–10 mg treatment group (pruritus at baseline: 16/25 [64%] vs 9/26 [35%] of patients). Similar findings were previously demonstrated in the phase III trial in PBC, where 39/70 (56%) and 50/73 (68%) of patients in the 5–10 mg and 10 mg treatment arms experienced pruritus compared to 28/73 (38%) in the placebo group. Reports of deaths in cirrhotic PBC patients linked with off-label (too high doses) OCA treatment prompted the FDA to issue a warning leading to intensified instructions for dose reduction in late-stage disease. As a note of caution, increased levels of fibroblast growth factor-19 (FGF19) following OCA treatment were observed in the AESOP trial, as anticipated by others, thus prudence regarding a potentially increased malignancy risk may be indicated particularly in PSC with its inherent high risk of cancer.

#### Non-steroidal FXR agonists

Concerns regarding adverse effects of OCA have fueled quests for alternative FXR agonists with maintained therapeutic effects such as decreased hepatic bile acids and (possibly) anti-inflammatory effects while avoiding adverse effects associated with OCA (pruritus, disagreeable lipid profile changes with HDL decrease and LDL increase, potential for drug-induced liver injury and hepatocarcinogenesis) [101].

Cilofexor, a non-steroidal FXR agonist, was investigated in a phase II trial (*n* = 52) with patients randomized to 12 weeks treatment with 100 mg (*n* = 22), 30 mg (*n* = 20) or placebo (*n* = 10) reporting that cilofexor was safe, well-tolerated and showed effect on biochemical endpoints (Table 3) [102]. Both the absolute and relative ALP reduction was larger in the 100 mg treatment arm compared to placebo (median ΔALP − 20.5% vs + 3.4%, *P* = 0.03), whereas significant differences were not seen for the 30 mg arm. In contrast to OCA, cilofexor did not induce pruritus, which occurred in 36% (8 of 22) of patients treated at 100 mg, 25% (5 of 20) at 30 mg, and 60% (6/10) with placebo.

Tropifexor is another non-steroidal FXR agonist with promising results in preclinical studies in non-alcoholic steatohepatitis (NASH) and currently under investigation in phase II studies in NASH and PBC (NTC02516605) [103, 104]. Along considerations above, tropifexor may hold therapeutic prospects in PSC, but currently no trials in PSC are listed for tropifexor at clinicaltrials.gov.

#### Aldafermin (NGM282)

FGF-19 is produced in the liver, gallbladder, and the distal small intestine upon FXR activation [92]. It inhibits bile acid synthesis via downregulation of CYP7A1 but as a note of caution may also stimulate cell proliferation in the liver and gut through pathways mediated by activation of IL6/STAT3 signaling [105], and has been implicated in the development of hepatocellular carcinoma in mouse models [106]. NGM282 is a synthetic analogue of FGF-19, which may provide beneficial effects in cholestatic liver disease through prolonged inhibition of bile acid synthesis. This FGF-19 analogue has been modified to enable biased FGFR4 signaling maintaining the regulatory functions of FGF-19 in bile acid metabolism while avoiding the STAT3 activation and hence lacking tumorigenic effects [107].

In an NGM282 phase II RCT including 62 patients with PSC, the primary endpoint of a significant decrease in ALP at 12 weeks was not met (ALP + 25.6 [+ 13.4%] and − 9.8 [− 2.8%] in the 1 mg and 3 mg treatment arms compared to − 0.6 for placebo; *P* = 0.43 and 0.65 for treatment groups compared to placebo) [107]. However, findings in exploratory endpoints might indicate improvement of hepatic injury and fibrosis (ΔELF test: − 0.3 for both treatment groups vs 0.1 for placebo, *P* = 0.049 and 0.02 for 1 and 3 mg compared to placebo; ΔPRO-C3: − 6.3, − 9.0 and 3.5 for 1 mg, 3 mg and placebo groups, *P* = 0.01 and 0.001 for 1 and 3 mg compared to placebo) and reduction in C4 (− 6.2 and − 9.4 ng/ml in 1 mg and 3 mg treatment groups) and bile acids (total endogenous bile acids: − 19.7, − 9.6 and − 4.1 in 1 mg, 3 mg and placebo groups; *P* = 0.16 and 0.04 for 1 and 3 mg compared to placebo).

Altogether, the multi-compound efficacy signals from activation of two distinct targets, FGF and FGF19, in the same biological pathway does hold promise for further trials in PSC, as they do for similar observations in PBC and NAFLD. The relationship between FXR/FGF19-signaling and changes observed in the gut microbiome in PSC also warrant clarification [17, 38, 108], and may even open for new treatment strategies in a disease in which inflammatory distribution overlaps almost perfectly with the enterohepatic circulation of bile acids [46, 109].

#### ASBT inhibitors

Further to the point on enterohepatic circulation of bile acids, ASBT is responsible for the reabsorption of conjugated bile acids in the terminal ileum. As mentioned, FXR exerts some of its effects through down-regulation of ASBT, reducing the enterohepatic circulation of bile acids and reducing the bile acid pool. Inhibition of ASBT is associated with improved liver histology in animal models of cholestatic liver disease and was hypothesized to bring therapeutic benefit in PSC [110–112]. An open-label phase II trial of an ASBT inhibitor (LUM001, maralixibat) has been completed with 27 PSC patients, and preliminary results available at clinicaltrials.gov indicate that no clinically relevant change in liver biochemistries was observed (Table 2). Concerns related to the potential impact from the increased colonic exposure of bile acids on diarrhea, colonic inflammatory activity or colonic dysplasia risk in PSC patients with IBD await further clarification.

#### All-trans retinoic acid

The cascades of events leading to transcriptional regulation by nuclear bile acid receptors FXR, PXR and VDR, mostly involves heterodimer formation with retinoid X receptor (RXR) as part of binding to relevant DNA response elements [67]. All-trans retinoic acid (ATRA) activates the nuclear receptor complex FXR/RXR, leading to reduced bile acid synthesis through SHP and potentially other pathways [113]. Promising findings in animal models of cholestasis provided the basis for studies evaluating ATRA as therapy for PSC [113, 114]. In a small pilot study (*n* = 15), combination therapy with UDCA (15–23 mg/day) and ATRA for 12 weeks did not meet the primary endpoint of 30% serum ALP reduction [median ΔALP − 34 (reduced from 277 to 243), − 12.3%, *P* = 0.09]; however, ΔALT − 39% (median ALT 76 vs 46, *P* = 0.001) (Table 2) [115]. A phase II trial investigating lower dose ATRA (10 mg b.d.) is ongoing (Table 3).

### Fibrates and PPAR activation

PPARs (PPAR-α in particular) are critical to the regulation of hepatic transporters involved in bile homeostasis and hence logical targets for therapy in cholestatic liver diseases. PPAR agonists have anti-cholestatic effects, including enhancement of biliary phospholipid secretion and mixed micelle formation through upregulation of MDR3, and inhibition of bile acid synthesis and upregulation of bile acid detoxification [116, 117]. Natural ligands include a broad variety of lipophilic acids, such as essential fatty acids, eicosanoids, phytanic acid, and palmitoylethanolamide [118]. As described for FXR, PXR and VDR, PPARs have pleiotropic effects, partially orchestrated by an intricate cross-talk with the bile acid nuclear receptors, including anti-inflammatory effects (e.g. by reduction in NF-κB-dependent gene activation and inflammatory cytokines), as well as anti-fibrotic effects (e.g. through reduced stellate cell activation and collagen deposition) [119]. In PBC patients with insufficient effect of UDCA, the pan-PPAR agonist bezafibrate demonstrated beneficial effects in a phase III investigator-initiated RCT [97, 120]. Patient-series indicate potential benefits from bezafibrate also in PSC [121, 122], but to date no RCT has been performed. Similar reports pertain to the PPAR-α agonist fenofibrate [123]. For the PPAR-δ agonist seladelpar, however, further trial activity in PSC (and NAFLD) has been suspended due to unexpected hepatic events (in the NAFLD arm).

The bezafibrate assessments in PSC originate from Japan (Table 2). A small prospective open-label study (*n* = 11, 12 weeks), showed promising results as assessed by reduced levels of ALP (by 54% at 12 weeks) and ALT with subsequent increase following cessation of treatment [122]. In a previous study, the same group reported effect (ALP reduced by 30.4% [median – 177] at 6 months) of bezafibrate 400 mg/day; ALP was reduced in 5/7 PSC patients [121]. In a small study of eight PSC patients only published as an abstract, fenofibrate was reported to induce significant reduction in ALP and ALT but no change in Mayo risk score at 6 months [124]. The larger French-Spanish study reported that fibrates (fenofibrate 200 mg/day or bezafibrate 400 mg/day for at least 6 months) in addition to UDCA in patients with ALP > 1.5 × ULN on UDCA alone, reduced ALP and ALT by 41% and 39% respectively at 3 months, as well as reduced pruritus in 20 PSC patients, with 40% of patients reaching ALP < 1.5 × ULN [123]. A multicenter RCT regarding the effect of bezafibrate on cholestatic itch in PBC and PSC started in 2016 and is still ongoing [125].

### Gut microbiota targeting therapy

The close association between PSC and IBD has long inspired hypotheses on PSC pathogenesis in which the bowel plays a causal role [126]. In Northern Europe and the USA almost 80% of PSC patients have proof of concurrent IBD, and since IBD in PSC is often right-sided and quiescent, the true number may be higher. In the opinion of the authors of this review article, the combined bowel-biliary inflammatory phenotype which predominates the patient pool of sclerosing cholangitis without an identifiable cause in Western countries, is likely to represent a single pathophysiological entity, with however variability in disease presentation and severity.

The mechanistic aspects of the role of the bowel in PSC are unclear. Whilst the earliest theories of PSC pathophysiology claimed a “leaky gut” whereby bacterial components and products would passively drift with portal blood to cause biliary inflammation, more recent sophistication involves specific interactions between the liver and the gut microbiome, and even “single-bug” associations (e.g. *Veillonella* and *Klebsiella*) [37, 40, 127]. Whether involvement of the gut microbiota relates to immunological stimulation, including innate immune activation, or bacterial metabolites of endogenous or exogenous origin, is unknown, as for the majority of diseases where the gut microbiome recently has been implicated [17]. Another fundamental question is whether observed alterations in the gut microbiota serve causal roles in the development of PSC or occur secondary to the ongoing disease processes, or both. The gut microbiota certainly holds strong co-metabolic functions in bile acid homeostasis [46, 128], and may influence bile physiology either directly or indirectly (e.g. via altered FXR signaling). How such metabolic influences compare with and potentially cross-talk with effects from the gut microbiota on the innate or adaptive immune system in PSC remains to be delineated.

Therapy directed at the gut microbiota is an attractive avenue to be explored in PSC. Modalities evolve rapidly along with general developments in the physiological elaboration of the gut microbiota, and cover broad and general approaches like non-absorbable antibiotics, diet and fecal transplantation, as well as more specific addition or deletion of specific bacterial strains through pro-/pre-biotics and bacteriophage applications, respectively [129, 130].

#### Antibiotic therapy

A number of antibiotics have shown suggestive ALP signals in PSC (Table 2), with the first reports appearing on tetracycline in 1959 and 1965, later also from metronidazole, azithromycin, vancomycin and minocycline (reviewed elsewhere [131]). Most emphasis has been put on vancomycin, which is a poorly absorbed (“gut-selective”) antibiotic shown to significantly modify the gut microbiota (particularly reducing *Bacteroides* and *Prevotella* species [132]), and may also hold immunomodulatory properties [133]. For vancomycin, there are three case-series and two small RCTs published and several studies registered as ongoing; however, long-term data are lacking and the total number of patients systematically investigated is still limited.

Initially, vancomycin therapy in PSC was reported in children, where ALP is not mentioned, as ALP is not used for follow-up of PSC in pediatric patients. The first case series from 1998 described three children with PSC and IBD who received vancomycin, all of which experienced reduced liver enzymes upon treatment with increased levels subsequent to drug withdrawal [134]. A patient series (*n* = 14) from 2008, observed improvement in ALT (*P* = 0.007) and GGT (*P* = 0.005) at 1–2 months in all 14 children, but less pronounced in cirrhotic patients [135]. At vancomycin discontinuation, liver enzymes increased in non-cirrhotic patients who then resumed treatment, resulting once again in reduced ALT and GGT. In 2013, vancomycin given to 14 children with PSC resulted in normalization of previously elevated GGT in all patients at 1–3 months, remaining normal after 12 months of therapy. Balancing these findings, a retrospective assessment of 66 children receiving vancomycin therapy in the registry of the Pediatric PSC consortium (22 as initial therapy and 44 after failing UDCA) [136], detected no statistically significant differences in liver-related outcomes between groups on vancomycin only (*n* = 22), UDCA only (*n* = 60), vancomycin after UDCA (*n* = 26), and no therapy (*n* = 35).

Two RCTs have been published for vancomycin in adult PSC patients (Table 2). In a small RCT (*n* = 35) from 2013, four groups of PSC patients (*n* = 8–9 per group) received vancomycin 125 or 250 mg four times per day or metronidazole 250 or 500 mg three times per day for 12 weeks. The primary endpoint of ALP reduction was achieved in both vancomycin groups (ΔALP in low- and high-dose vancomycin: − 43%, *P* = 0.003 and − 40%, *P* = 0.002) but not in the metronidazole groups [137]. Results assessed by other endpoints (bilirubin, Mayo risk score) were conflicting showing improvement in one or more low-dose groups only and not in high-dose groups. In another RCT from 2016, vancomycin was compared to placebo (*n* = 29; *n* = 18 vs 11 for vancomycin vs placebo groups), reporting an effect on ALP reduction at 3 months compared to 1 month of treatment [ΔALP − 142.9 (− 18.2%), *P* = 0.02 in the treatment group, compared to ΔALP − 58.1 (− 6.6%) in placebo group] [138]. Slight differences in baseline ALP should however be noted (1160 versus 897 in treatment versus placebo groups, respectively).

For an open-label phase III trial of vancomycin in PSC, results are available at clinicaltrials.gov (ClinicalTrials.gov identifier: NCT01802073) but not published for 9/14 children and 9/20 adults who completed the study. The main reasons for non-completion were non-compliance in children (4/5) and physician’s decision in adults (7/11). Change in ALP was not a primary outcome and data are not given. In adults, 6/9 (66.7%) and 6/9 (66.7%) achieved each of the two primary outcomes of clinically significant reduction in ALT and GGT, respectively, at 3 months, compared to 100% (10/10 and 8/8 for ALT and GGT) of children. At 2-year follow-up, there were no deaths or serious adverse events in adults (0/20) and only 1/14 children experienced a serious adverse event (stricture requiring stenting). For an open-label study of vancomycin in PSC and biliary atresia, results are available at clinicaltrials.gov (ClinicalTrials.gov identifier: NCT01322386) but not published for 9/11 enrolled children 0–16 years of age with PSC. Some PSC patients (*n* = 11) signed consent but were not enrolled. Improved liver blood tests (not specified which and no levels given) were reported for 9/9 PSC patients.

Metronidazole and UDCA combination therapy was compared to UDCA + placebo in PSC patients (*n* = 80, 36 months) in a RCT, resulting in decreased ALP (ΔALP − 337 vs − 214, i.e. − 52.4% vs − 37.7%), and Mayo risk score. Furthermore, significantly more patients showed histologic improvement of stage (34%, *P* = 0.047) or grade (34.4% vs 14%, *P* = 0.014) in the metronidazole group [139]. An open-label pilot study for rifaximin in PSC (*n* = 16, 12 weeks) failed to show significant changes in ALP (ALP increased with 5 IU/mL from 342 to 345; + 1%) and rifaximin was concluded to lack effect [140]. Minocycline has several properties in addition to being antibiotic, potentially contributing to its therapeutic effects in PSC, including anti-inflammatory (e.g. upregulation of the potent anti-inflammatory cytokine IL-10) and antiapoptotic properties [141, 142]. An open-label pilot study of minocycline (1 year) showed significant improvement of ALP (ΔALP − 65, 19.7%; *P* = 0.04) and Mayo score [143].

In sum, the promising efficacy signals from long-term antibiotic treatment in PSC provide proof-of-concept support for the notion of a pathogenic role of the gut microbiome. However, as for the overall changes in the gut microbiota in PSC, further studies are needed to clarify the mechanisms responsible for the observations. Delineation of these mechanisms may open new avenues for more specific therapies involving the gut microbiome in PSC, potentially overcoming the troublesome issues related to side effects and promotion of anti-microbial resistance that occurs with gross antibiotics therapy. As of yet, long-term antibiotics prescription in PSC should only be performed in the context of clinical trials.

#### Fecal transplantation

Fecal transplantation (FMT) is established therapy for recurrent *Clostridium difficile* colitis refractory to treatment with antibiotics, for which it has demonstrated clinical benefits [144]. Enthusiasm has been high regarding the use of FMT for treatment of PSC [17] and IBD [145]; however, interpretation of current data in the field is complicated by the substantial variations in current protocols as to route and frequency of application, stool preparation prior to transplantation, choice of donors, randomization and well-defined control groups [146]. For PSC, only one full-text paper has been published to date reporting on the effects of FMT in PSC [41]. This open-label phase I/II study investigated the safety and efficacy of a single FMT delivered by colonoscopy in 10 patients with PSC and concomitant IBD. At week 24 post-FMT, the predefined endpoint of ≥ 50% ALP reduction from baseline was reached by 3/10 (30%, or more specifically 3/9) and there were no adverse events. The change in ALP is not numerically reported in the article, but graphical presentation per patient shows fluctuating levels of ALP over time suggesting that at 12 weeks there would be no overall difference from baseline. The abundance of engrafter operational taxonomic units in patients post-FMT correlated with decreased ALP levels (*P* = 0.02) whereas no significant effect of FMT on bile acids was found.

#### Probiotics and bacteriophage applications

Probiotic therapy was investigated in PSC patients with a cocktail containing four *Lactobacillus* and two *Bifidobacillus* strains in a small crossover RCT (*n* = 14, 3 months) [147]. Results showed no difference in ALP (− 9 vs. − 9%; *P* = 0.99), other liver enzymes or symptoms. A phase III trial is registered (NCT00161148) but has surpassed its completion date without status verification for several years.

A pathobiont is a commensal bacterium, which may shift from symbiosis (a symbiont) to exerting pathogenic features depending on the circumstances. The suggestions of *Veillonella* and *Klebsiella pneumonia* involving in PSC pathogenesis seem convincing [37, 40, 127], however the chicken-and-the-egg problem remains open, as both microbes seem to be involved in other liver and non-liver diseases and may also appear abundant due to the presence of advanced liver disease. The concept however is interesting, as it opens for the targeting of specific bacteria, as successfully done in murine experiments by specific bacteriophage killing in alcoholic hepatitis and PSC [130, 148]. As our understanding of the gut microbiota in human diseases evolves, we are likely to see more specific therapeutic approaches along this thinking, targeting specific bacteria or specific pathways of gut microbial physiology.

### Immune modulating therapy

It is a paradox in PSC that all immune-modulating therapies so far have failed to be effective in halting disease progression despite strong genetic and circumstantial data implying that PSC is an autoimmune disease [18–25]. Furthermore, T-cells dominate the portal inflammatory infiltrates in liver biopsies in PSC [149], partially resulting from cholangiocyte-immune cell cross-talk [31, 32], partially also possibly through migration of activated lymphocytes from the gut to the liver (“homing”) as stated by the “aberrant T-cell homing”-hypothesis on PSC pathogenesis [26, 27]. Even during heavy immunosuppression following liver transplantation, PSC patients develop disease recurrence at relatively high frequencies [150], contrasting the immune and autoimmune signatures that should suggest otherwise.

#### Glucocorticoids: immunosuppression in PSC with features of autoimmune hepatitis

There is a continuous spectrum of autoimmune activity across PSC patients, with higher activities typically seen in the young. In patients with PSC fulfilling the diagnostic criteria for autoimmune hepatitis (AIH; 7–14% of adult patients with PSC), traditionally denominated “PSC-AIH overlap”, more recently diagnosed as “PSC with features of AIH” [151], glucocorticoid therapy along standard guidelines for treatment of autoimmune hepatitis is recommended although not evidence based [152]. Treatment response is often less pronounced than in autoimmune hepatitis alone, and evaluation of effects on transaminases and IgG must be weighed against corticosteroid side effects (e.g. osteoporosis) and discontinuation may be considered in patients in whom there is little impact.

There is no evidence for clinically significant efficacy of glucocorticoids in PSC patients without AIH [153] or in PSC with elevated IgG4 unless a diagnosis of IgG4-associated sclerosing cholangitis can be made per the HISORt criteria [154]. Observational data are discouraging. A cohort study (*n* = 21) reported significant, but marginally important, improvement in serum ALP (ΔALP − 23%; *P* = 0.003) and AST levels (119 ± 14 vs 103 ± 19 U/L, *P* = 0.02) at 1 year following budesonide therapy with loss of effect 3 months post-treatment, with no effect on Mayo risk score or histology and a significant loss of bone density as an important side effect [155]. No significant effect on liver biochemistries was observed in a RCT (*n* = 12, 24 months) [156] or another RCT comparing budesonide to prednisolone (*n* = 19) [157].

#### Azathioprine, ciclosporin, tacrolimus, methotrexate, and mycophenolate

For other immunosuppressants, very limited data exists. Studies are generally few and mostly lack placebo or are small (*n* = 2–30) and underpowered. Although ALP reduction has been reported in singular cases following treatment with azathioprine, mycophenolate mofetil (MMF) and tacrolimus, proof of any effect on clinical outcome is lacking.

Due to the association of PSC with IBD, many patients are taking azathioprine for their IBD at the time of PSC diagnosis and progression. There are no RCTs for azathioprine as PSC therapy. Existing data are limited to case reports and a prospective case series (*n* = 15, median 41 months of treatment) [158], and although improvement in ALP (mean ΔALP − 557 [− 55.8%]; ALP normalized in 5/15 patients) and histology (6/10, 2/10 and 2/10 showed improved, stable or worsened histology, respectively) was reported in this series, there were no convincing data of effect on clinical outcomes. Of note, a Swedish population based registry study have suggested an impact from azathioprine (and statins) on clinical outcomes in PSC [159], so further and more targeted assessments to this end may still be considered.

Ciclosporin inhibits IL-2 transcription and hence T-cell response and possibly T regulatory cell production, while methotrexate exerts anti-inflammatory properties through suppression of T-cell activation and adhesion molecule expression. One placebo-controlled trial (*n* = 35) investigated ciclosporin in PSC patients but was aimed at evaluating effect on UC activity, and does not report ALP change, other liver biochemistries or liver histology [160]. For methotrexate, a preliminary trial showed promising improved histology at 1 year in 6/9 patients, but a RCT (*n* = 24, 2 years) by the same group found no effect on liver biochemistry or histology [161]. However potential bias was reported by the authors, with baseline cirrhosis slightly more frequent in the treatment group compared to placebo (58% vs 42%) [162]. Any effect on clinical outcome has not been proven, but it may be argued that it has not been well investigated for the two drugs.

Regarding MMF, a potent immunosuppressant that attenuates both B- and T-cell proliferation, a pilot study (*n* = 30) showed a significant but clinically marginal ALP reduction (ALP reduced by 223 [19.6%] from 1135 to 912, *P* = 0.02) [163]. Subsequently, a small RCT did not show improved effect of combination therapy with MMF (1 g/day) + UDCA (*n* = 12) over UDCA alone (*n* = 13) following 2 years of treatment (ALP change − 16.3% vs + 2.8% in MMF vs placebo groups at 2 years; histology and cholangiography also showed no effect of treatment) [164]. For tacrolimus, which inhibits IL-2 receptor expression and IL-2 production and hence T-cell proliferation, two small open-label trials (*n* = 10 and 16, respectively) have been published [165, 166], both showing effect on liver biochemistry. The former showed 70% reduction in ALP at 1 year, concluding that tacrolimus will be an important agent in the treatment of PSC [166]. In the larger of these, ALP was reduced by 46.5% in the *n* = 8 (50%) patients who completed 1 year of therapy [165]; however, drop-out numbers were high due to poor tolerance for the drug, perhaps due to gastrointestinal side effects associated with a high number of proctocolectomized patients.

#### Biologics in the treatment of PSC

Infliximab is a monoclonal antibody inhibiting TNF-α, commonly used in the treatment of severe IBD. In PSC, one small pilot study was conducted in which infliximab failed to demonstrate any effect on ALP, histology or liver related symptoms (Table 2) [167]. Currently, an ongoing international multicenter retrospective study initiated by the International PSC Study Group (IPSCSG) is investigating the effect on ALP and PSC-related clinical events of anti-TNF-α therapy used for IBD in PSC patients. Preliminary reports from the assessment do not suggest an efficacy on the hepatobiliary disease in PSC from anti-TNF therapy [168].

Vedolizumab, a selective humanized monoclonal antibody to the α_4_β_7_ integrin expressed on lymphocytes, blocks gut lymphocyte trafficking through inhibition of the binding of α_4_β_7_ integrin to MadCAM-1. In the bowel, this leads to reduced intestinal inflammation and induction of mucosal healing and vedolizumab has emerged as an effective treatment option in refractory IBD [169, 170]. During inflammatory conditions, PSC included, MadCAM-1 and relevant chemokines (e.g. CCL25, CCL28 and CXCL12) are detected in the portal areas [171–173], suggesting vedolizumab may represent a putative therapeutic agent in PSC. However, results of a small retrospective study (*n* = 34) and subsequently a larger international retrospective study (*n* = 102) in patients with PSC and IBD have been disappointing, showing no evidence for significant improvement of liver biochemistry in PSC [174, 175]. Whether a dedicated trial of vedolizumab in PSC will be performed, is thus doubtful.

VAP-1 is an adhesion molecule expressed by hepatic endothelial cells which supports leukocyte recruitment to sites of inflammation through NF-κβ dependent expression of other adhesion molecules including MadCAM-1 expression on endothelial cells [176]. VAP-1 is highly expressed in PSC livers compared to healthy livers [177], particularly on the endothelial lining of sinusoids, but also on fibrous septa in cirrhotic PSC livers. A phase II, single arm, open-label, multicenter clinical trial BUTEO is investigating the safety and activity in PSC of timolumab, a VAP-1 inhibitor (BTT1023, a fully humanized monoclonal antibody against VAP-1), but results are not yet ready (Table 3).

#### Anti-inflammatory and anti-fibrotic therapies

Cenicriviroc is a CCR2/CCR5 antagonist with anti-inflammatory and antifibrotic effects demonstrated in animal models of fibrosis and NAFLD [178]. Final results are still pending for a 24 week phase II trial for cenicriviroc in PSC, but provisory results posted on clinicaltrials.gov indicate that ALP was reduced but not normalized following treatment (mean ALP reduction − 4.5% at 24 weeks; no patients achieved ALP normalization or 50% ALP reduction but 50% [*n* = 10] of patients achieved ALP reduction to < 1.5 × ULN). In NAFLD, cenicriviroc is one of the compounds being taken to phase III stage of evaluation, and is also scrutinized in combination with the FXR agonist tropifexor (see above) as one of the first attempts at multi-targeted, combination therapies in this entity [179]. The latter point is interesting also from a conceptual perspective, summarizing the knowledge from clinical trials in PSC reviewed within the present chapter: is a single target sufficient? Or will multiple pathways ultimately have to be addressed for efficacy to be expected, e.g. bile acid, immune targets and gut microbiota targets jointly—each with small, but additive effects?

Simtuzumab is a monoclonal antibody directed against lysyl oxidase-like 2 (LOXL2), a matrix enzyme which plays a central role in fibrogenesis, stabilizing the fibrotic matrix by catalyzing cross-linkage of elastin and type I collagen. LOXL2 activity induction was reported in fibrotic liver diseases including PSC and hepatic and serum LOXL2 levels correlated with fibrosis in PSC [56, 180, 181]; hence LOXL2 appeared to be an attractive target for therapy. In a phase II clinical trial, patients with compensated PSC (*n* = 234; half of which had bridging fibrosis or cirrhosis at baseline) were randomized to treatment with simtuzumab 75 mg, 125 mg or placebo for 96 weeks [57]. Results were disappointing, however, for the primary endpoint, showing no effect on fibrosis as assessed by hepatic collagen content. Results were also negative for both treatment arms compared to placebo regarding Ishak fibrosis scores and event-free survival. Overall, 80 (34%) patients had fibrosis progression (34%, 33% and 44% in the 75 mg, 125 mg and placebo groups) and 47 (20%) experienced PSC-related clinical events.

The simtuzumab trial is nevertheless interesting, due to its opportunities for exploratory endpoints considerations against liver biopsy findings. In total, 47 patients (20%) experienced at least one of the clinical events included in the co-primary endpoint (ascending cholangitis [*n* = 31, 13%], jaundice [*n* = 15, 6%], ascites [*n* = 7, 3%], sepsis [*n* = 4, 2%], CCA [*n* = 3, 1%]); hepatic encephalopathy, variceal hemorrhage and hepatocellular cancer each with < 1%) but no deaths or liver transplantations occurred, underscoring the need for good surrogate endpoints. In multivariate analysis, PSC-related clinical events were more frequent in patients with (at baseline) advanced fibrosis (hazard ratio [HR] 2.03; 95% confidence interval [CI] 1.02–4.06; *P* = 0.045), higher ALP (HR per 10 U/L, 1.01; 95% CI 1.00–1.02; *P* = 0.015), and higher ELF test (HR per unit, 1.26; 95% CI 0.98–1.61; *P* = 0.073).

Stratification of patients by baseline ALP demonstrated a stepwise increase in risk of clinical events at week 96 (risk 6%, 19% and 34% in patients with baseline ALP < 158, 158–324 and > 324 U/L) [57], supporting previous reports of an association between ALP > 1.5 × ULN with clinical outcomes in PSC. However, change in ALP at week 12 or other time points were not associated with clinical events in the simtuzumab trial.

### Study design and biomarkers

Successful development of therapy in PSC is currently hampered by uncertainties on study design and lack of prospectively validated surrogate markers for efficacy. Examples of challenges related to study design include: (1) long disease duration and low event-rate; (2) lack of early stage disease identifiers; (3) confounding comorbidity, IBD in particular; (4) high disease variability and fluctuating disease activity; and (5) lack of established biomarkers for (a) risk stratification and clinical trial patient selection and (b) as surrogate markers for disease severity and treatment effects.

A majority of the clinical trials conducted in PSC to date have been ill powered. This is mainly due to the combination of a rare disease with slow disease progression (in some patients over decades) where definitive outcomes such as liver transplantation or death rarely occur within a relatively short study period. Furthermore, clinical assessment of large patient series support the existence of sub-groups of patients with distinct prognostic features (e.g. PSC with UC is associated with more aggressive disease compared to PSC with Crohn’s disease) [3]. Refined and more stringent phenotype definitions of PSC patients entered into trials might thus contribute to improved power. The high frequency of cholangiocarcinoma and other malignancies in PSC is also a challenge, representing both a confounder for disease behavior and potentially relevant endpoints.

Improved selection of patients based on disease severity or stage would enhance our ability to conclude reliably from trials. Furthermore, the fluctuating disease course in PSC is poorly captured by currently used severity measures and prognostic algorithms, over and above the transient aggravations that may occur related to intercurrent events such as biliary obstructions and bacterial cholangitis. Finally, one may even argue that at the time where a diagnosis of PSC is currently made, i.e. based on biliary strictures on imaging, it is already too late for many of the proposed mechanisms of action for relevant drugs to be efficacious. Such considerations lead to deep questions on how to make a diagnosis of PSC, and whether current emphasis on imaging may lead to an intrinsic underrepresentation of early stages where medical therapy may be expected to show efficacy, i.e. the biliary strictures as an irreversible feature—by some denominated “cirrhosis of the bile ducts”.

#### Alkaline phosphatase

ALP is the single marker receiving most attention in clinical trials in PSC and was used as a primary or secondary endpoint in almost every clinical trial in PSC with published data over the past 20 years (Tables 1, 2). Most studies of therapy in PSC have also used elevated ALP at baseline as one of the inclusion criteria. The International PSC Study Group presented expert opinion in a position paper based on a Delphi process of reiterated discussions, concluding that ALP is one of the top five candidate surrogate markers for clinical trials in PSC [61]. In a follow-up paper, authors affiliated with the U.S. Food and Drug Administration (FDA) also favors ALP as a surrogate endpoint, although concluding that ALP should be supported by other biomarkers or clinical benefit [60]. They base their conclusion on a claim that a limited pool of published data support that ALP normalization, or reduction by 40%, or achieving a level less than 1.3–1.5 × ULN may identify patients with improved outcomes [182–185]. The arguments in favor of using ALP as a primary endpoint are that (1) ALP reflects disease severity in PBC and could be expected to perform similarly in PSC; (2) ALP increases with increasing symptoms caused by significant biliary strictures and decreases following their treatment in observational studies; and (3) four retrospective cohort studies reported that normalization of ALP or improvement to < 1.3–1.5 × ULN was associated with better clinical outcome [182–185]. As a note of caution, though, prospective data are lacking and two of these retrospective studies were long-term post hoc analyses of UDCA trials.

In the 10-year follow-up study for the Scandinavian medium–high-dose UDCA trial, patients who achieved ALP normalization or 40% reduction or reduction to 1.5 × ULN had improved transplant-free survival regardless of treatment arm (UDCA or placebo) [8]. In the high-dose UDCA trial (*n* = 150), ALP reductions were not related to complications in individual patients in the UDCA treatment arm [182]. Rather, evidence suggesting an association of ALP with prognosis in this trial rests on the fact that patients who achieved ALP ≤ 1.0 × ULN at least once during follow-up (in the high-dose UDCA as well as the placebo arm) had a lower risk of death and LT compared to patients in the same treatment arm who did not experience ALP normalization at any point.

In summation, it is reasonable to say that most arguments in favor of ALP are weakly founded and further studies to clarify its utility are certainly warranted. Is ALP truly a suitable surrogate disease severity marker or risk stratification tool in PSC? The cutoff values for ALP at baseline and the definition of change in ALP (magnitude and time point for evaluating change) varies between different studies (Tables 1, 2). Hence, the algorithms proposed to date also generally lack external validation and attempts at cross-validation have failed (e.g. ALP < 1.5 × ULN was discriminatory at 2 years in one cohort [185] but was only predictive when applied at 6 or 12 months in two other studies [184, 186], which did not replicate this cutoff value at 2 years). The considerations hold considerable importance, as exemplified by ALP efficacy signals in several recent phase II clinical trials (*nor*UDCA: ΔALP − 12.3%, − 17.3% and − 26.0% compared to placebo in the 500 mg, 1000 mg and 1500 mg treatment groups; OCA: ΔALP − 25.7%; bezafibrate: ΔALP − 40%; fenofibrate: ΔALP − 43%; vancomycin: ΔALP − 18.2% to − 43%), which at best parallel past experiences with UDCA (ΔALP − 40.6% to − 72.6%), and hence should prompt reflections as to whether these signals indeed will translate into clinical benefit—and as such fundamentally also how to deal with UDCA prescription in PSC.

The challenges related to patient inclusion based on ALP can be exemplified by a panel extracted from the PSC patient registry in Oslo, where one or more ALP values were available for a random selection of 112 PSC patients. Depending on whether the lowest or highest ALP value represented a hypothetical time of screening for a clinical trial, 30 (26.8%) or 26 (23.2%) patients had ALP values within the normal range, while 48 (42.9%) or 41 (36.6%) patients had ALP < 1.5 × ULN, hence failing to comply with a commonly used cut-off point for inclusion in clinical trials. Bilirubin levels exceeded 2.0 mg/dL in 24 (21.4%) of patients at both time points. Applying the combined criteria of ALP > 1.5 × ULN and bilirubin ≤ 2.0 mg/dL, only 42 (37.5%) and 71 (43.8%) of the patients would be eligible for inclusion. Thus, from about a quarter to more than half of our patients would be excluded from several ongoing or past clinical trials in PSC contributing to the power issues of clinical trials in PSC, and moreover, might lead to a selection bias that may hamper generalization of results.

#### Other surrogate endpoints

A recent review of surrogate endpoints in PSC endorsed by the FDA as well as the previous position paper on the same subject by the International PSC Study Group both give much space to the discussion of ALP [60, 61]. Acknowledging, however, the challenges associated with ALP as a prognostic biomarker in PSC, the development of other non-invasive tests has received intense attention over recent years (Table 4). Several PSC-specific clinical models, comprising a mixture of clinical features and laboratory values, and based on logistic regressions or machine-learning methodology, have been suggested to serve prognostic purposes yet frequently include ALP [187, 188]. The prognostic scores may turn out to represent useful surrogate endpoints, although the most widely used clinical model, the revised Mayo risk score, demonstrated limited utility in early stage disease and failed to predict the negative outcome of the high-dose UDCA trial. Certainly, their broad scope of parameters may also render them less sensitive to dynamic changes within distinct pathways.

**Table 4:** Suggested surrogate markers for therapeutic trials in PSC

Biomarker	Pathophysiologic target	PRO	CON
*Non-invasive: serum based tests and clinical scores*			
ALP	Cholestasis Inflammation	Consistent association with clinical outcome in multiple studies [182–185, 198]. Reflects biliary inflammation and cholestasis, thus biologically meaningful	Fluctuates naturally during the course of PSC, thus single measurements in individuals are unreliable Diverging definitions of defined risk groups or treatment effect across studies (various cutoff values proposed; or 40% reduction in ALP; or normalization); attempts at cross-validation of suggested definitions have failed
Amsterdam-Oxford model	Clinical score; PSC subtype, age at PSC diagnosis, albumin, platelets, AST, ALP, bilirubin	Showed adequate discriminative performance and good prediction accuracy at PSC diagnosis and during follow-up; independently validated in a large (*n* > 500) international study [187, 199]	Dynamic features and responsiveness to various therapies have not been tested Some components are not modifiable by therapy
Autotaxin		Consistent association with shorter transplant-free survival in two independent panels in a monocenter study as well as with 2.6-fold risk of liver transplantation or death in another study [195, 200]	Variation over time in individuals not tested
Bilirubin	Cholestasis	Consistent and strong association with clinical outcome in multiple studies. Directly reflects cholestasis, thus biologically meaningful	Intercurrent increases may be due to temporary and treatable bile duct obstruction by gallstones or sludge, bacterial cholangitis, or strictures available to endoscopic therapy. Increasing bilirubin due to liver failure is a late event in PSC and not useful as a surrogate marker in early disease
CD14	Gut barrier function: bacterial translocation	Associated with transplant-free survival in a monocenter study, independent of Mayo score [33]	Independent validation is lacking Variation over time in individuals not tested
ELF test	Fibrosis A direct test of fibrogenesis based on a panel of fibrosis markers (HA, PIIINP, TIMP-1)	Strong association with clinical outcome (LTX or death) independently of the Mayo risk score; initially shown in two independent panels at a single center, then independently validated in a large, international multicentre study [189, 190] Showed change at 12 weeks of treatment in a phase II study on NGM282, indicating dynamic potential [107] ELF test directly reflects fibrogenesis, which is important in the pathogenesis of PSC and a therapeutic target	Variation over time in individuals not tested. Usefulness in early disease not tested
IL-8	Inflammation	Associated with clinical outcome in PSC as a serum marker (and in bile) [194]. May reflect biliary inflammation as a clinically meaningful biological pathway	Outperformed by fibrosis markers regarding prediction of transplant-free survival
Mayo risk score	Clinical score; age, bilirubin, AST, INR, variceal bleeding status	Strong association with clinical outcome; the most commonly used clinical risk score in PSC	Not validated at the individual level. Did not predict adverse events in a previous high-dose UDCA trial INR and variceal bleeding and partly bilirubin reflect changes seen in advanced disease Some components are not modifiable by therapy
PREsTo	Clinical score; age, years since PSC diagnosis, bilirubin, albumin, ALP x ULN, platelets, AST, hemoglobin, sodium	Associated with clinical outcome defined as hepatic decompensation. Derived using machine-learning techniques in a large (*n* = 509) multicenter North American panel and validated in an international multicenter cohort (*n* = 278) [201]	Validation in an independent study is lacking Some components are not modifiable by therapy
PRO-C3	Fibrosis A specific marker of collagen III formation	Associated with clinical outcome (LTX or death) in a single center study [193] Showed change at 12 weeks of treatment in a phase II study on NGM282, indicating dynamic potential [107] PRO-C3 directly reflects collagen III formation, which is an important part of fibrogenesis and hence the pathogenesis of PSC, and a therapeutic target	Usefulness in early disease not tested
PRO-C5	Fibrosis A specific marker of collagen V formation	Associated with clinical outcome (LTX or death) in a single center study [193]	Usefulness in early disease not tested
VAP-1	Autoimmunity Leukocyte recruitment to sites of inflammation	Vap-1 predicted clinical outcome in two independent PSC patient panels from two different centers in one study [177]	No independent validation study No prospective data
*Non-invasive: imaging*			
Transient elastography (TE)	Fibrosis Liver stiffness as a proxy for fibrosis	Baseline LSM values and ΔLSM were both associated with clinical outcome in a retrospective, single center study, and validated in a study from an independent center [191, 192]	Impact of severe cholestasis/cholangitis uncertain Not applicable in ascites or (severe) obesity
MR elastography (MRE)	Fibrosis Liver stiffness as a proxy for fibrosis	Associated with clinical outcome (hepatic decompensation) in a large (*n* = 266), single center retrospective study [202]	Not widely available Costly. Time-consuming
Annali score (MRCP)	MRC findings of intrahepatic bile duct dilatation, dysmorphy (lobar atrophy, lobular surface changes, or an abnormal caudate to right lobe volume ratio) and portal hypertension	Annali score without gadolinium was associated with clinical outcome in a single center study, then validated in two independent panels in a large, international, multicentre study [203, 204]	Major weight on late changes related to cirrhosis Dynamic changes are likely to be slow
*Invasive*			
Histological stage	Fibrosis Grading systems may include various pathophysiologic processes	Strong association with clinical outcome (LTX-free survival, time to LTX) for stage assessed by Ishak, Nakanuma, and Ludwig staging systems was demonstrated in a single-center study and validated in an independent international multicentre study. Nakanuma staging appeared to have the best prognostic value [205, 206] Association of Ludwig stage with clinical outcome has been demonstrated in several studies One study estimated (using a Markov model) that change in stage at 2 and 5 years would appear in 66% and 96% of PSC patients with Ludwig’s stage II at baseline, indicating that changes in histology can be seen within the scope of a therapeutic trial [207]	Invasive, risk of adverse events Staging discord between multiple biopsies unless care is taken to biopsy same localization (using ultrasound) No consensus on a single system for histological grading and staging of PSC No data to support the use of change in histological grade as a surrogate marker The most prominent pathology in PSC relates to the larger bile ducts, which are not accessed by standard liver biopsy
Biliary calprotectin	Biliary inflammation	Associated with transplant-free survival in two studies by independent groups [194, 208]. Biologically meaningful: calprotectin is expressed by neutrophils, activated monocytes and macrophages and acts as a chemotactic molecule, reflecting biliary duct inflammation as a parallel to the use of fecal calprotectin in inflammatory bowel disease	Requires invasive sampling by ERCP, with risk of adverse events

*ALP* alkaline phosphatase, *AST* aspartate transferase, *CCA* cholangiocarcinoma, *ELF* Enhanced Liver Fibrosis test, *ERCP* endoscopic retrograde cholangiopancreatography, *HA* hyaluronic acid, *IL-8* interleukin-8, *LSM* liver stiffness measurements, *LTX* liver transplantation, *MRCP* magnetic retrograde cholangiopancreatography, *MRE* magnetic resonance elastography, *PIIINP* propeptide of type III procollagen, *PSC* primary sclerosing cholangitis, *TE* transient elastography, *TIMP-1* tissue inhibitor of metalloproteinases-1, *VAP-1* vascular adhesion protein-1

Tests reflecting liver fibrosis seem promising. The patented and commercially available serum-based Enhanced Liver Fibrosis (ELF) test and ultrasound based transient elastography have shown strong associations with clinical outcome in PSC independent of other factors, and have both been validated in independent studies from different centers or in multicenter cohorts [57, 189–192]. Novel and more specific biomarkers of liver fibrosis, discriminating collagen formation from degradation as opposed to just reflecting the more static fibrosis load, have also been associated with clinical outcome in PSC and may be suited to evaluate treatment effects, with PRO-C3 the currently most promising single marker [193]. Markers of inflammation, autoimmunity or gut barrier function including IL-8 [194], VAP-1 [177], autotaxin [195] or soluble CD14 [33] have also demonstrated association with clinical outcome and may represent relevant surrogate endpoints in trials targeting these pathways; however, independent validation and more knowledge about the natural history and fluctuations of theses markers, alone and in combination, are warranted.

## Conclusions

The role of UDCA in the treatment of PSC remains controversial. A surge of interest in clinical trials in PSC over recent years has resulted in proof-of-concept for bile acid therapies beyond UDCA using novel agents as well as antibiotic treatment directed at the gut microbiota (Fig. 1). Antifibrotics and therapy targeting the immune system seem paradoxically disappointing. The establishment of validated risk stratification tools and surrogate endpoints would facilitate drug development. Likely, combination therapy covering several aspects of PSC pathogenesis will ultimately be needed to achieve clinical efficacy.

## Abbreviations

AE2: Chloride/bicarbonate anion exchanger type 2
ALP: Alkaline phosphatase
ASBT: Apical sodium-dependent bile acid transporter
CCA: Cholangiocarcinoma
CFTR: Cystic fibrosis transmembrane conductance regulator
GWAS: Genome-wide association studies
FDA: The US Food and Drug Administration
FGF19: Fibroblast growth factor 19
FXR: Farnesoid X receptor
IBD: Inflammatory bowel disease
MadCAM-1: Mucosal vascular addressin cell-adhesion molecule 1
MRC: Magnetic resonance cholangiography
MRI: Magnetic resonance imaging
OCA: Obeticholic acid
PPAR: Peroxisome proliferator-activated receptor
PBC: Primary biliary cholangitis
PSC: Primary sclerosing cholangitis
RCT: Randomized clinical trial
UDCA: Ursodeoxycholic acid
